# Ecological Specialization to Fluctuating Resources Prevents Long-Distance Migratory Raptors from Becoming Sedentary on Islands

**DOI:** 10.1371/journal.pone.0061615

**Published:** 2013-04-23

**Authors:** Laura Gangoso, Pascual López-López, Juan Manuel Grande, Ugo Mellone, Rubén Limiñana, Vicente Urios, Miguel Ferrer

**Affiliations:** 1 Department of Wetland Ecology, Estación Biológica de Doñana, CSIC, Seville, Spain; 2 Vertebrate Zoology Research Group, CIBIO Research Institute, University of Alicante, Alicante, Spain; 3 Centro para el Estudio y la Conservación de las Aves Rapaces en Argentina (CECARA)/Instituto de Ciencias de la Tierra y Ambientales de La Pampa (INCITAP)- CONICET, FCEyN, UNLPam, La Pampa, Argentina; 4 Instituto de Investigación en Recursos Cinegéticos (IREC), CSIC-UCLM-JCCM, Ciudad Real, Spain; 5 Department of Ethology and Biodiversity Conservation, Estación Biológica de Doñana, CSIC, Seville, Spain; Institut Pluridisciplinaire Hubert Curien, France

## Abstract

**Background:**

The adaptive transition between behavioral strategies, such as the shift from migratoriness to sedentariness, remains an outstanding question in evolutionary ecology. Density-dependent variation in the age of first breeding has been proposed as a feasible mechanism through which long-lived migratory birds with deferred sexual maturity should become sedentary to persist on islands. Although this pattern seems to hold for most raptors and herons, a few exceptions have been identified. One of these exceptions is the Eleonora’s falcon, a long-distance migratory bird, which shows one of the most peculiar adaptations in the timing of reproduction and food requirements among raptors.

**Methodology/Principal Findings:**

Here, we compiled data concerning demography, banding recoveries and satellite tracking of Eleonora’s falcons to discuss likely explanations for the exceptional behavior of this insular long-distance migratory species.

**Conclusions/Significance:**

New data reveal that Eleonora’s falcons do return to the natal colonies in their first year and young birds are able to breed. However, in contrast to previous hypothesis, the highly specialized strategy of this and other ecologically similar species, as well as the virtual lack of food during winter at breeding areas prevent them from becoming sedentary on islands. Although the ultimate mechanisms underlying the process of sedentarization remain poorly understood, the evidence provided reveal the existence of important trade-offs associated with ecological specialization that may become particularly relevant in the present context of global change.

## Introduction

All organisms that live in seasonally changing environments must adjust their life cycles to maximize fitness. Migration is probably one of the most remarkable adaptations of animals to seasonality. Migration, however, also entails important costs in terms of energy expenditure and survival, and a delayed arrival may represent the loss of prior occupancy advantages [1]. These costs may be critical for juvenile individuals of long-lived species with deferred sexual maturity, for which the risk of migration may outweigh the advantages of gaining experience on breeding performance during their first years of life [2].

Although migratory behavior is mainly under genetic control [3], great variation in the appearance/disappearance and magnitude of this complex phenomenon occurs among and within species over surprisingly short time scales. Thus the evolution of migratory behavior in a resident population or of residency in a migratory one may be a common and rapid process driven by appropriate selection pressures [3], [4]. However, variation in migratory behavior may also be environmentally determined. Phenotypic flexibility can be advantageous when colonizing new areas and may be especially important for understanding the number of phenotypic shifts that generally accompany the successful colonization of islands [5]. On islands, the improved survival resulting from milder climates, year-round food availability, and fewer predators/competitors, may favour sedentary behavior and hence, a loss of migration is likely to arise [6].

Recently, Ferrer and coauthors [7] proposed a mechanism by which long-lived birds become sedentary on islands. The authors reviewed the migratory status of two groups of large birds with deferred sexual maturity, namely Accipitriformes and Ciconiiformes. They found that, in contrast to the pattern in passerine species, almost all Accipitriformes and Ciconiiformes are sedentary on islands, and that they switch to this behavior even if they are migratory on the mainland. Density-dependent variation in the age of first breeding can be crucial for the long-term survival of small isolated populations of long-lived species [7]. Ferrer and colleagues argued that since the probability of occupation of vacancies in the breeding population depends on the ability of potential breeders to detect them, the buffering effect of the variation in age of first breeding may be evident in sedentary populations, but not in migratory ones with delayed return, i.e., individuals do not return to breeding areas in their first year of life, but wait until the second or third year to reach these areas with a new status of potential breeder.

Nevertheless, the aforementioned authors state that three species of raptors and two species of herons breed on islands but have not become sedentary. The Eleonora’s falcon (*Falco eleonorae*) is one of these exceptions to the hypothesis. In particular, this species contradicts the predictions that 1) “the only migratory species able to colonize islands are those without deferred sexual maturity”, and 2) “those species with deferred sexual maturity must be sedentary, even if the mainland populations are, in fact, migratory”. In this regard, [7] suggested that the lack of delayed return could be a likely explanation to account for these exceptions.

Here, we discuss the abovementioned predictions in respect to life-history traits of the Eleonora’s falcon. To this end, we compiled data from the literature and our own field research concerning demography, banding recoveries and satellite tracking of individual birds.

## Results and Discussion

The Eleonora’s falcon is a medium-sized long-distance migratory raptor that breeds colonially on islands distributed throughout the entire Mediterranean basin and winters mainly in Madagascar [8]–[12]. The bulk of the breeding population is concentrated in the Eastern Mediterranean Sea, with only a few colonies located in the Atlantic Ocean, including those on the Canary Islands which represent the westernmost and southernmost populations of its entire breeding range [13]. The Eleonora’s falcon shows a very particular ecology, not only for its long-distance trans-equatorial migration, but also for its precise linkage between foraging behavior and the timing of reproduction [13]. Indeed, it is the latest breeder (from mid-August to late October) of all raptor species breeding in the Northern Hemisphere.

Contrary to the first prediction, this species presents deferred sexual maturity; the modal age at first breeding for Eleonora’s falcons is usually two (3^rd^ calendar-year) for females and three (4^th^ calendar-year) for males [14]. Our data from the Canary Islands partially confirm this pattern, given that the youngest recorded breeders (considering only birds banded as nestlings that were resighted in a territory) were three 3^rd^ calendar-year females but also six 3^rd^ calendar-year males (3.12% and 6.25% of the breeding population, respectively, n of pairs = 99) paired in 2009 and ten 3^rd^ calendar-year females and three 3^rd^ calendar-year males (11.90% and 3.57%, respectively, n = 84) paired in 2011. Notwithstanding, there are a few exceptions to the generality with some 2^nd^ calendar-year birds paired, often with older birds, although not raising juveniles [14], [15]. In the Canary Islands, two 2^nd^ calendar-year females initiated courtship with adult males but they did not lay eggs in 2010, while in 2011 three 2^nd^ calendar-year females were paired with adult males with the same outcome. In addition, we observed another 2^nd^ calendar-year female, which replaced the missing partner of an adult male at the end of the breeding season.

It has been suggested that the Eleonora’s falcon, despite being highly phylopatric [16], [17], shows delayed return. However, long-term studies seem to contradict, at least in part, this assumption. Data from satellite-tracked Eleonora’s falcons from Spain, Italy and Greece demonstrate that 2^nd^ calendar-year birds (n = 6) do in fact leave the wintering grounds during spring, heading northwards [8], [18], [19]. Although many of them may not necessarily reach their natal colonies the first year [20], satellite telemetry data, banding recoveries and observations show that these young birds disperse widely throughout the Mediterranean basin [14], [17], [18], [20], thus becoming distributed across potential future breeding sites during their first summer. For instance, the only 2^nd^ calendar-year individual satellite-tracked during its first full spring migration, wandered around mainland Spain and France until September, before it finally visited its natal colony on the Columbretes Islands late in the breeding season [18]. Furthermore, on the basis of banding recoveries, [16] reported six 2^nd^ calendar-year individuals re-sighted 50–500 km (n = 3) and >500 km (n = 3) away from their natal colony in Greece. Our data also confirmed that a fraction of Eleonora’s falcons banded as nestlings in the Alegranza Islet (Canary Islands) returned to their natal areas in their 2^nd^ calendar-year: 6.7% of the nestlings banded in 2010 were re-sighted in the natal colony in 2011 (n = 165). In addition, three 2^nd^ calendar-year birds banded in Alegranza were re-sighted on other islands of the archipelago, and another three inland on peninsular Spain. The percentage of 2^nd^ calendar-year birds returning to their natal colonies may however vary locally and among years (e.g. from 0 to 47.6% and 0–3% of the aged birds on Sicily and Crete, respectively [14], [15].

If not to breed, why do 2^nd^ calendar-year birds return to their natal breeding colonies?

Although long migratory journeys are a costly part of a bird’s life [2], previous experience may allow skilled birds to return earlier and more rapidly to their breeding grounds in following years, when reproduction is a more likely reward. At the same time, these birds may learn feeding, courtship and breeding behaviors from the adults that may be crucial for their successful breeding in subsequent years. Furthermore, inter-annual variation in resource availability at breeding grounds might provide them the opportunity to reproduce in exceptionally good years. In accordance with this hypothesis, our data on breeding performance on the Canary Islands show high inter-annual variability in clutch size (Levene test F_4,428_ = 15.05, *p*<0.0001, n = 433) and productivity (Levene test F_4,441_ = 4.41, *p* = 0.0017, n = 446), suggesting that individuals of different quality, including immature and/or inexperienced birds, might be joining the breeding fraction of the population in some years. Interestingly, the proportion of 2^nd^ calendar-year birds recorded in Salina (Sicily) increased while the number of breeding pairs and the average breeding success declined, and some of these young birds (90% females) attempted to breed paired with adult individuals [15].

Therefore, although it has been suggested that long-distance migratory birds show lower rates of phenotypic change than short- and medium-distance migrants [4], the compiled data also suggest an apparent flexibility in migratory behavior as well as the age of first breeding. Both aspects would partially support the hypothesis proposed by [7]. In this respect, Eleonora’s falcons would behave like other long-lived species that have become sedentary on islands, i.e. buffering the occasional population fluctuations thus allowing for the long-term persistence of populations.

Well then, what explains the fact that Eleonora’s falcons have not become sedentary on islands? During the breeding season these falcons feed primarily upon the influx of migratory birds heading to Africa. Consequently, breeding colonies are strategically situated in small and desolated islands located along the main migratory routes [13]. The influx of this resource may vary widely both within and among years, given that it strongly depends upon wind patterns [21] (L.G. & J.M.G. unpub. data). However, by the end of October this superabundant resource definitely ceases, thus elapsing an extended period without food during the winter months. Although Eleonora’s falcons are somehow phenotypically flexible and can shift their diet from migrant passerines to insects in wintering areas [12], [13], breeding grounds remain practically barren throughout the rest of the year, even with respect to insects [20] (L.G. & J.M.G. unpub. data). As a consequence, it is plausible that the lack of sufficient food for maintaining a dense population (this species breeds colonially at extremely high densities for a *Falco* of its size [13]) could prevent its shift to residency on islands. In accordance with this supposition, the Rough-legged buzzard (*Buteo lagopus*), which constitutes another exception to the hypothesis proposed by [7], may illustrate a similar example. This Holarctic species is considered as an irruptive migrant, i.e., these birds perform year-to-year flexible migratory movements following peaks of prey populations, primarily voles and lemmings [2], which remain hidden by deep snow during the long northern winters. Therefore, although the species also shows some flexibility in its migratory behavior and age of first breeding [22], unfavorable environmental conditions at breeding grounds during the winter and, especially, its high level of specialization to a fluctuating food resource, may have precluded residency of Rough-legged buzzards, not only on islands, but also on the mainland.

All of this evidence reveals the existence of important trade-offs associated with ecological specialization to a fluctuating resource. In the case of the Eleonora’s falcon, this specialization has allowed for the widespread colonization of the Mediterranean basin, where this species can exploit almost exclusively a temporally superabundant food resource (i.e. small migratory birds) to raise offspring, while avoiding inter-specific competition during spring (the season in which most other bird species breed in the Northern Hemisphere). Conversely, Eleonora’s falcons, due to their high level of specialization, are forced to travel long distances (up to 10,000 km), thus facing the inherent high costs in terms of potential mortality twice a year. Moreover, this specialist species may be particularly susceptible to incidental variations associated with climate change in the phenology of their prey populations, such as advances or delays in their migration schedules [23], as well as potential changes in wind regimes. Furthermore, the inability to shift towards sedentarism on islands may bring about important evolutionary consequences. In contrast to sedentary populations, evolutionary adaptation and genetic divergence could be slowed or even hindered in these migratory populations. Therefore, we conclude that, although migration itself represents an important cost related to the high level of ecological specialization to a food resource, the risk of phenological mismatch due to climate change may become an additional cost as a result of the extreme specialization of long-lived species.

## Materials and Methods

We compiled data from the literature and our own field research concerning demography, banding recoveries and satellite tracking of individual birds.

### Ethics Statement

Corresponding permissions were granted by the Spanish Regional Administrations (“Dirección General del Medio Natural, Consejería de Medio Ambiente y Ordenación Territorial, Gobierno de Canarias”, and “Dirección General de Gestión del Medio Natural, Conselleria de Medi Ambient, Aigua, Urbanisme i Habitatge, Generalitat Valenciana”, Spain), according to the Spanish law “Ley 42/2007 de 13 de diciembre del Patrimonio Natural y la Biodiversidad”. Specific permissions numbers: MAOT N° 11908, MAOT N° 6468, MAOT N° 9723, E-87-10-T and E-59-11-E. Based on the relevant legislation (“Real Decreto 1201/2005”, and “ley 32/2007 de 7 de noviembre para el cuidado de los animales, en su explotación, transporte, experimentación y sacrificio”), an ethical approval is not required for this study. Capture and tagging of birds did not require specific approval of the Animal Care and Use Committee (IACUC) or ethics committee. This legislation was developed from the regulation of the European Council (Agreement ETS 123) of the European Union (Council Directive 86/609/CEE).

### Field Procedure

**Figure 1:**
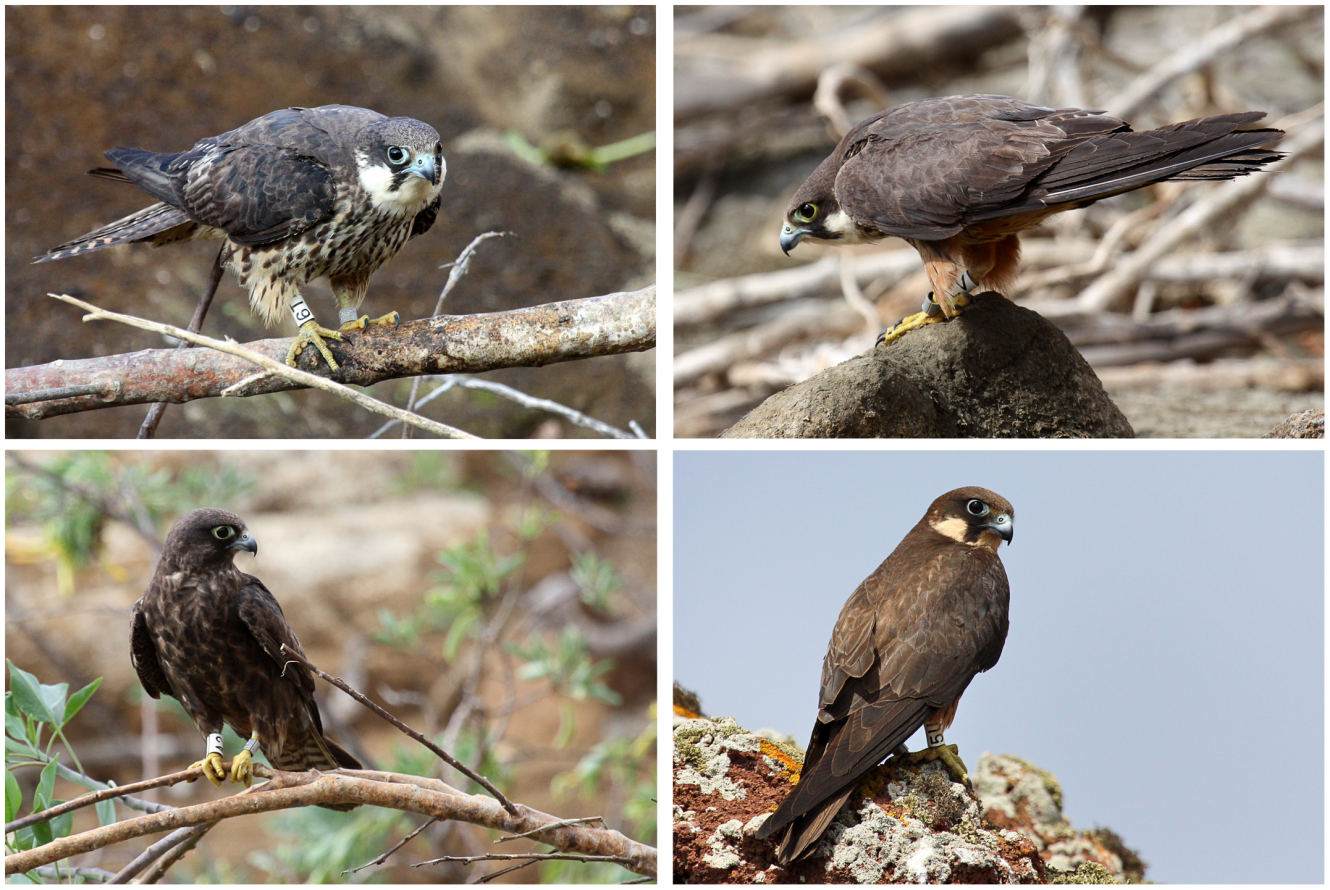
Second calendar-year Eleonora’s falcons banded as nestlings in 2010 re-sighted in the natal colony (Alegranza Islet) in 2011. (Top left) pale female, (top right) pale male, (bottom left) dark male. (Bottom right) Breeding pale female banded as nestling in 2007 that breeds successfully since 2009, re-sighted in 2011.

The Eleonora’s falcon population from the Alegranza Islet (10.5 km^2^, 289 m a.s.l.), Canary Islands (27°37′–29°25′N, 13°20′–18°19′W) was intensively monitored during the period 2006–2011. The islet is privately owned and has been protected under the category of Natural Park since 1994. This colony comprises ca. 120 breeding pairs, which represent 45% of the pairs breeding in the Archipelago (pers. obs.). Thus far, a total of 964 Eleonora’s falcons (775 nestlings plus 189 adults) have been banded. Adult birds were trapped using dho-gaza nets and a stuffed Eagle Owl (*Bubo bubo*) as a decoy. Nestlings were marked at nests at the age of 20–25 days. All birds were banded with a numbered aluminum ring and released after manipulation. Additionally, in 2007 (140 nestlings and 52 adults, 10 of them recaptured birds banded in previous years) individuals were also tagged with a combination of three colored metal rings. In 2009 (167 nestlings and 26 adults, 11 recaptured), 2010 (165 nestlings and 34 adults, 11 recaptured) and 2011 (145 nestlings and 22 adults, 3 recaptured), two-digit plastic rings were used, allowing for long-distance identification via terrestrial telescopes and photographs (see Fig. 1). Re-sights were conducted at a pond where birds regularly bath and drink, and at nests.

### Satellite Tracking

A total of 18 Eleonora’s Falcons were trapped on the Balearic and Columbretes Islands (Spain) in autumn between 2007 and 2010. Both regions have been protected under the category of Natural Park since 1995 and 1998, respectively. Birds were equipped with Microwave Telemetry Inc. 9.5-gram solar-powered satellite transmitters. Transmitters were programmed to collect data on a duty cycle of 12 h on/58 h off. Locations were collected using the Argos system. For this study we only used summer data of one immature (2^nd^ calendar-year) male born on the Columbretes Islands in 2010 for which we recorded the complete summering event, from arrival into the Mediterranean basin until the onset of the following autumn migration (see [10]–[12] for details on trapping methods and data processing).
